# Pollen extract in association with vitamins provides early pain relief in patients affected by chronic prostatitis/chronic pelvic pain syndrome

**DOI:** 10.3892/etm.2014.1861

**Published:** 2014-07-24

**Authors:** TOMMASO CAI, FLORIAN M.E. WAGENLEHNER, LORENZO GIUSEPPE LUCIANI, DANIELE TISCIONE, GIANNI MALOSSINI, PAOLO VERZE, VINCENZO MIRONE, RICCARDO BARTOLETTI

**Affiliations:** 1 Department of Urology, Santa Chiara Regional Hospital, Trento, Italy; 2 Clinic and Polyclinic for Urology, Child Urology and Andrology, University Hospital of Giessen und Marburg, Justus-Liebig University, Giessen, Germany; 3 Department of Urology, University Federico II, Naples, Italy; 4 Department of Urology, University of Florence, Florence, Italy

**Keywords:** chronic pelvic pain syndrome, pollen extract, chronic prostatitis symptom index, quality of life, prostatitis syndrome

## Abstract

The therapeutic efficacy for chronic prostatitis/chronic pelvic pain syndrome (CP/CPPS) is currently unsatisfactory. The aim of the present study was to assess the safety and efficacy of pollen extract in association with vitamins (DEPROX 500^®^) in males with CP/CPPS. All patients with a diagnosis of CP/CPPS attending the same urologic centre between March and October 2012 were enrolled in this randomised controlled phase III study. Participants were randomised to receive oral capsules of DEPROX 500^®^ (two capsules every 24 h) or ibuprofen (600 mg, one tablet three times a day) for four weeks. The National Institutes of Health Chronic Prostatitis Symptom Index (NIH-CPSI), International Prostate Symptom Score and Quality of Well-Being (QoL) questionnaires were used. In the intention-to-treat analysis, 87 males (25 class IIIa and 62 class IIIb) with a mean age of 33.6±5.9 years were randomly allocated to the DEPROX 500^®^ (n=41) or ibuprofen (n=46) treatment groups. At the follow-up examination (following one month of treatment), in the DEPROX 500^®^ group, 31/41 patients (75.6%) reported an improvement in quality of life, defined as a reduction of the NIH-CPSI total score by ≥25%, compared with 19/46 (41.3%) in the control group (P=0.002). The greater improvement in the DEPROX 500^®^ group compared with the ibuprofen group was statistically significant (treatment difference in the NIH-CPSI pain domain, −2.14±0.51, P<0.001; QoL scores, P=0.002). All patients were negative at the Meares-Stamey test evaluation. Adverse events were less frequent in the DEPROX 500^®^ group than in the ibuprofen group. The DEPROX 500^®^ treatment significantly improved total symptoms, pain and quality of life compared with ibuprofen in patients with CP/CPPS, without severe side-effects.

## Introduction

Chronic prostatitis (CP) has been described as one of the most common illnesses in males aged <50 years (1), and exhibits different clinical presentations (2). According to the classification of the National Institutes of Health (NIH) (3), class III CP/chronic pelvic pain syndrome (CP/CPPS) is the most frequent category (4), in which either genitourinary symptoms or pain are usually found and the impact on quality of life is considerable (5). The efficacies of current therapies for CP/CPPS are unsatisfactory (6). Phytotherapeutics are a noteworthy option due to their generally minimal side-effects; however, few have been subjected to scientific scrutiny and prospective controlled clinical trials (7,8). In previous years, a number of studies have shown that pollen extract preparations are able to yield a durable and marked reduction of symptoms in young males with CP/CPPS, with an improvement in semen quality and a significant reduction in the NIH-Chronic Prostatitis Symptom Index (CPSI) score (9–11). Previously, Wagenlehner *et al* demonstrated that a standardized pollen extract significantly improved the total symptoms, pain and Quality of Well-Being (QoL) scores in patients with inflammatory CP/CPPS without severe side-effects, highlighting the role of the anti-inflammatory activity of pollen extract (12). In the last year, Cai *et al* demonstrated that pollen extract in association with vitamins significantly improved the total symptoms, pain and QoL scores in patients with non-inflammatory CP/CPPS without severe side-effects in a phase II study (10). Furthermore, the association with vitamins is likely to improve the antioxidant activity of the pollen extract as well as the protective effect on nerves and also reduce the pain in patients with inflammatory or non-inflammatory CP/CPPS (10). The aim of the present study was to assess the safety and efficacy of pollen extract in association with vitamins in comparison with ibuprofen in order to improve the quality of life of patients affected by CP/CPPS by the relief of pain.

## Materials and methods

### Study design

In order to assess the safety and efficacy of pollen extract in association with vitamins (DEPROX 500^®^) in males with CP/CPPS, all consecutive patients with a clinical and instrumental diagnosis of CP/CPPS (class IIIa or b), attending the same urologic centre (Santa Chiara Regional Hospital, Trento, Italy) between March and October 2012 were screened for this prospective randomised controlled phase III study. The design of the study was in accordance with the guidelines for clinical trials in CP/CPPS described by the NIH Chronic Prostatitis Collaborative Research Network (13). No placebo arm was included. The possible biases caused by the lack of placebo arm were considered in the results analysis. No placebo run-in period was considered necessary due to the fact that all enrolled patients were not blinded. The main outcome measure was the improvement of quality of life at the end of the whole study period, defined as the symptomatic improvement in the pain domain of the NIH-CPSI. Clinical failure was defined as the persistence of low quality of life following the treatment (failure to obtain a reduction of the NIH-CPSI total score by ≥25%), or the suspension of therapy for significant reported adverse effects (12). In addition, spontaneously reported adverse events, or those noted by the investigator, were recorded during the whole study period. The study was conducted in line with Good Clinical Practice guidelines, with the ethical principles laid down in the latest version of the Declaration of Helsinki. Written informed consent was obtained from all patients prior to treatment. Furthermore, this study was conducted in line with the Consolidated Standards of Reporting Trials statement (The Ottawa Hospital Research Institute, Ottawa, ON, Canada).

### Study schedule

**Figure 1:**
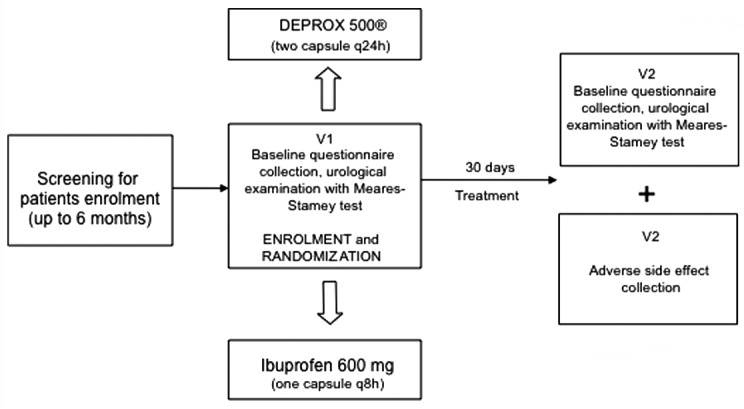
Study schedule. V1 = baseline, V2 = follow-up visit at 30 days. q, every.

On arrival at the center, all eligible individuals provided their written informed consent and underwent baseline questionnaires, urological examination and the Meares-Stamey test that was performed by the same urologist in accordance with the procedure described in the European Association of Urology (EAU) guidelines (14). All patients who met the inclusion criteria undertook oral administration of DEPROX 500^®^ (two capsules every 24 h) or ibuprofen (600 mg, one tablet three times a day) for four weeks. Ibuprofen was selected in accordance with the results obtained by Lee *et al* (15). Proton-pump inhibitors (PPIs) were not routinely used due to the fact that all patients with gastrointestinal bleeding or a history of duodenal or gastric ulcers were excluded. Enrolled patients were not blinded to the preventative treatment. All patients were assigned to the two groups (DEPROX 500^®^ and ibuprofen) according to a 1:1 randomization (Fig. 1). All patients were contacted by telephone on day 14 of the therapy to ensure correct timing and dose of treatment. Each subject was scheduled for a follow-up examination at 30 days from starting therapy, with a urological and microbiological examination and questionnaire collection.

### Inclusion and exclusion criteria

Inclusion criteria were the presence of symptoms of pelvic pain for at least three months during the six months before study entry, according to the EAU guidelines, a score in the pain domain of the NIH-CPSI (14) of >7 and a negative four-glass result in the Meares-Stamey test (12). Subjects <18 and >65 years of age, affected by major concomitant diseases, with known anatomical abnormalities of the urinary tract or with evidence of other urological diseases, and with residual urine volume >50 ml resulting from bladder outlet obstruction were excluded. Males with a reported allergy to pollen extract, who had recently (<4 weeks) undergone oral or parental treatment or who were currently using prophylactic antibiotic drugs were also excluded. Additionally, all patients with a history of gastrointestinal bleeding or duodenal or gastric ulcers were excluded. All patients positive to tests for *Chlamydia trachomatis* (Ct), *Ureaplasma urealyticum*, *Neisseria gonorrhoeae*, herpes viruses (HSV 1/2) and human papillomavirus (HPV) were also excluded.

### Composition and characterization of the extracts used

All patients who were randomized to the DEPROX 500^®^ group underwent oral administration of two tablets of DEPROX 500^®^ in a single dose daily in the evening, in line with our previous study (10) and with the manufacturer’s instructions (IDI Integratori Dietetici Italiani S.r.l, Sicily, Italy). Each administration contained 1 g pollen extract (500 mg per tablet), and vitamins B1, B2, B6, B9, B12 and PP. All compound analyses were carried out in accordance with the procedures described by Fiamegos *et al* (16). All patients randomized to the ibuprofen group received ibuprofen (600 mg) three times per day.

### Questionnaires and urological examinations

The validated Italian versions of the NIH-CPSI (17) and the International Prostate Symptom Score (IPSS) (18) questionnaires were administered to each patient. The questionnaires were self-administered when the patient arrived at the urologic centre. Furthermore, patient quality of life was measured by using an Italian version of the QoL scale, a validated, multi-attribute health scale (19). This scale was selected because it has been successfully applied to acute illnesses, whereas other quality of life scales, including the Short Form-36 (SF-36) Health Survey, are more suitable in chronic cases (20). Higher scores on the QoL scale reflect a higher quality of life. In accordance with the study by Nickel *et al* (21), prostatitis-like symptoms were considered significant at a pain score of ≥4. The NIH-CPSI was also used in determining clinical therapy efficacy (21).

### Sample collection and laboratory procedures

All samples were collected during the urological examination and immediately taken to the laboratory, under refrigerated conditions, analysed for cultures and aliquoted for DNA extraction and polymerase chain reaction for Ct, *Neisseria gonorrhoeae*, HSV 1/2 and HPV detection. All subjects included in the study underwent urinary culture for common bacteria, yeasts and urogenital mycoplasma. Microbiological culture was carried out in accordance with the methods described by Mazzoli *et al* (22). DNA extraction and purification from urine were performed using the EZ1 DNA Tissue kit (Qiagen SpA, Milan, Italy), as described in our previous study (22).

### Statistical analysis

The primary target of the study was the symptomatic improvement in the pain domain of the NIH-CPSI. In order to analyse the homogeneity of the two groups, the baseline characteristics were compared using the Student’s t-test and Mann*-*Whitney U test for continuous variables and by the χ^2^ test for categorical variables. The normal distribution of the variables was assessed using the Kolmogorov-Smirnov test. Data were analysed based on the intention-to-treat (ITT) approach. General characteristics of the study participants were expressed using descriptive statistics (means, standard deviations and ranges). The required sample size for the present study was calculated under the following conditions: Difference between the groups, 2±1 score points in the NIH-CPSI pain domain; α error level, 0.05 two-sided; statistical power, 80%; and anticipated effect size, Cohen’s d=0.5. The calculation yielded 2×39 individuals per group. Randomization based on a single sequence of random assignments (simple randomization) was performed using a pseudo-random number generator software (Research Randomizer Version 4.0, Social Psychology Network, Wesleyan University, Middletown, CT, USA). Analysis of variance (ANOVA) was used for comparing the means. The Bonferroni adjustment test was also used at the second stage of the ANOVA. The effect size between the means (Cohen’s d) was also calculated. The differences between the groups regarding the NIH-CPSI results were obtained using an ANOVA test. Statistical significance was achieved when P<0.05. All reported P-values were two-sided. Statistical analyses were performed using SPSS software, version 11.0 (SPSS, Inc., Chicago, IL, USA) for Apple-Macintosh.

## Results

### Patients

**Figure 2:**
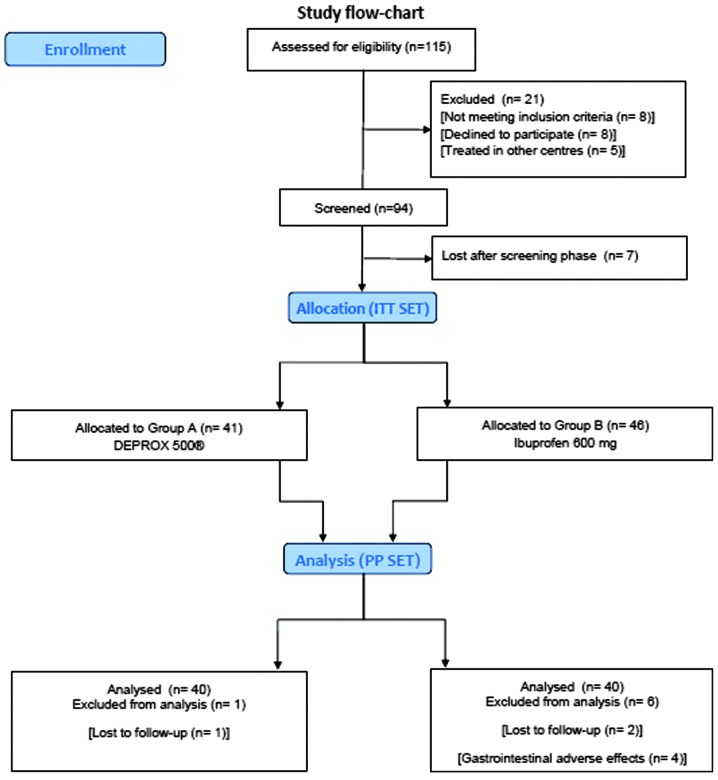
Study flow-chart according to the Consolidated Standards of Reporting Trials statement. ITT, intention-to-treat; PP, per protocol analysis.

From the 115 patients attending the center for prostatitis-like symptoms during the study period, 94 were eventually enrolled and randomised. Out of the 21 patients excluded from the study, eight refused to be enrolled, six reported adverse effects to nonsteroidal anti-inflammatory drugs, two reported a clinical history of gastrointestinal ulcers and five elected to be treated in other centres. Additionally, seven patients were lost subsequent to randomisation and 87 males were finally enrolled (Fig. 2). The baseline questionnaire mean scores were 25.9±2.1, 8.0±3.6 and 0.55±0.15 for NIH-CPSI, IPSS and QoL, respectively. Historical medical information and clinical data at enrolment are described in Table I. No statistically significant differences between the groups were identified.

### Randomisation

Of the 87 enrolled patients (mean age 33.6±5.9 years), 41 received DEPROX 500^®^ (group A), and 46 received 600 mg ibuprofen (group B). The treatment arms were comparable for all variables at the enrolment and randomisation visits.

### Compliance with treatment schedule and adverse effects

In group A, 1/41 patients (2.4%) had mild adverse effects that did not require additional treatment (nausea), while in group B, 7/46 patients (15.2%) reported nausea and epigastric pain. In group A, 40 patients (97.5%) were analysed subsequent to one being lost in follow-up. In group B, 38 patients (82.6%) were analysed subsequent to two being lost to follow-up and four discontinuing therapy due to gastrointestinal adverse effects. The DEPROX 500^®^ treatment was well tolerated in all the patients analysed, and no significant drug-related side-effects were identified. The analyses were carried out in the ITT (pollen extract, n=41; ibuprofen, n=46) and per protocol (PP) populations (pollen extract, n=40; ibuprofen, n=40) (Fig. 2).

### Clinical and laboratory results at follow-up (after one month of treatment)

**Figure 3:**
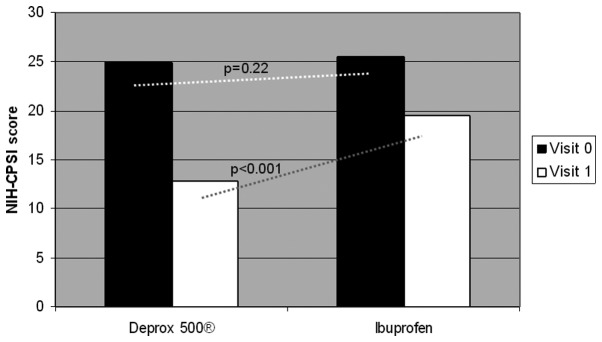
Statistically significant differences between the two visits in terms of the NIH-CPSI scores (P<0.001) between the two groups. NIH-CPSI, National Institutes of Health-Chronic Prostatitis Symptom Index.

**Figure 4:**
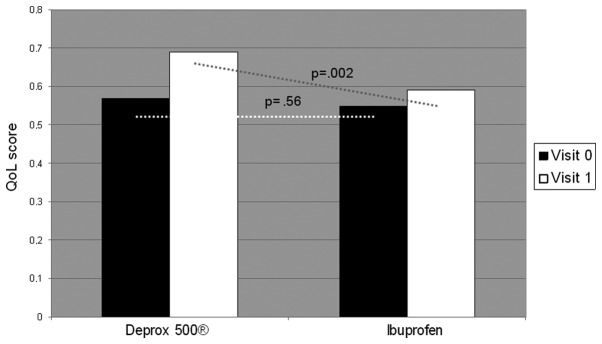
Statistically significant differences between the two visits in terms of the QoL scores (P=0.002) between the two groups. QoL, Quality of Well-Being.

At the follow-up examination, in the ITT set and DEPROX 500^®^ group, 31/41 patients (75.6%) reported an improvement of quality of life, defined as a reduction of the NIH-CPSI total score by ≥25%, compared with 19/46 (41.3%) in the control group (P=0.002). In the PP set and DEPROX 500^®^ group, 31/40 patients (77.5%) reported an improvement of quality of life, compared with 20/40 (50.0%) in the control group (P=0.019). The questionnaire results at one month after treatment were as follows: NIH-CPSI, 12.8±2.20; IPSS, 7.6±1.58; and QoL, 0.69±0.10 in the DEPROX 500^®^ group. By contrast the results in the control group were: NIH-CPSI, 19.5±2.10; IPSS, 8.00±2.81; and QoL, 0.59±0.18. The higher improvement in the DEPROX 500^®^ group compared with the ibuprofen group was statistically significant (treatment difference in NIH-CPSI pain domain: ITT, −2.14±0.51, P<0.001; PP, −1.76±0.22, P<0.001). Statistically significant differences were also reported in the NIH-CPSI (P<0.001), and QoL (P=0.002) scores between the two visits in the DEPROX 500^®^ group and between the two groups (Figs. 3 and 4). No statistically significant differences were identified in the IPSS scores (P=0.43). All patients were negative at the Meares-Stamey test evaluation. All questionnaire results at the follow-up visit are presented in Table II. The results of the physical examinations, including vital signs, and laboratory examinations showed no relevant changes from the baseline.

### Sub-analysis on the basis of CP/CPPS type

Of the 87 patients, 25 (28.7%) showed inflammatory CP/CPPS (type IIIa), while 62 (71.3%) exhibited type IIIb. A statistically significant difference was identified between the two groups in terms of pain relief and QoL improvement when stratified by CP/CPPS type. In fact, in the DEPROX 500^®^ group, patients affected by type IIIb CP/CPPS showed higher QoL results and a lower pain level following treatment (the NIH-CPSI score was 24.8±1.8 at the enrolment versus 11.7±1.7 at the follow-up visit; P<0.001) when compared with type IIIa CP/CPPS patients (Table III). No differences were reported between the ITT or PP sets.

## Discussion

The major finding of the present study was that DEPROX 500^®^ is able to provide early pain relief and improve the quality of life in patients with CP/CPPS without severe side-effects, when compared with ibuprofen. Furthermore, it was revealed that patients affected by type IIIb CP/CPPS may obtain greater advantages from this therapy. These findings lead to several points of discussion. Firstly, the early pain relief. In 2006, Elist (23), using a double-blind study with random distribution versus placebo, demonstrated the superiority of pollen extract versus placebo in terms of improvement in pain score and the filling and emptying symptoms from the start to the end of the treatment after six months of therapy. Additionally, in 2009, Wagenlehner *et al* (12) showed that pollen extract improved symptoms, pain and quality of life after 12 weeks of treatment in patients with this condition, with differences in favour of pollen extract at six weeks of treatment compared with the placebo, and the treatment being well tolerated. These two studies treated the patients for at least six weeks (12,23). Consistent with our previous study (10), 30 days of treatment with DEPROX 500^®^ in the present study was able to provide significant results in terms of pain reduction when compared with ibuprofen. This effect is possibly due to the association between the pollen extract and vitamins B6 and B12 that improve the antioxidant activity of pollen extract with the protective effect on nerves. B vitamins including thiamine (B1), pyridoxine (B6) and cyanocobalamin (B12) are capable of antinociception in experimental animals with acute and chronic pain evoked by electrical, chemical and thermal stimulation, primary neuronal injury and diabetes (24,25). Notably, several studies have demonstrated that certain B vitamins, particularly B6 and B12, are able to protect neurons from certain injuries (26,27). The B vitamins, B1, B6 and B12, are clinically useful in the treatment of certain painful conditions including lumbago, sciatica, trigeminal neuralgia and chronic pain associated with diabetic polyneuropathy (28). Finally, we hypothesized that the early improvement on pain relief is due to the protective effect on nerves, and the following improvement in quality of life could be due to the antioxidant activity of pollen extract. Indeed, previous studies in which only pollen extract was administered demonstrated an improvement in quality of life and pain relief after ≥6 weeks of treatment. Furthermore, on the basis of the sub-analysis, the patients that best obtained the important advantages from this therapy were those with non-inflammatory CP/CPPS. Contrasting with previous studies, the present study revealed that patients with non-inflammatory CP/CPPS showed improved results compared with those with inflammatory CP/CPPS. This is possibly due to the fact that the protective effect on nerves of B vitamins occurs earlier than the anti-inflammatory effect of pollen extract. In this sense, DEPROX 500^®^ is able to provide improved results in terms of early pain reduction in patients with non-inflammatory CP/CPPS. DEPROX 500^®^ was generally well tolerated over the full study period.

The present study had a few limitations that should be taken into account: The small number of enrolled patients, a short follow-up period, a selected patient population, the lack of control group and that this was not a blinded study. Given the lack of prove efficacy of conventional therapies, alternative treatment options are urgently required and pollen extract in association with vitamins should be an noteworthy option due to its generally low side-effects and promising results in terms of quality of life improvement.

In conclusion, given the aforementioned limitations, DEPROX 500^®^ significantly improved the total symptoms, pain, and quality of life compared with ibuprofen in patients with CP/CPPS, without severe side-effects.

## Figures and Tables

**Table I:** Baseline characteristics and clinical parameters at enrolment.

Parameter	DEPROX 500^®^ group	Ibuprofen group
Patients, n	41	46
Age, years^a^	33.8±6.78	33.7±5.44
Marital status, n (%)		
Married	19 (46.3)	18 (39.1)
Unmarried	22 (53.7)	28 (60.8)
Educational qualification, n (%)		
Primary school	5 (12.2)	7 (15.2)
High school	29 (70.7)	27 (58.6)
University	7 (17.1)	12 (26.2)
Smoker status, n (%)		
Yes	11 (26.8)	13 (28.2)
No	30 (73.2)	33 (71.8)
Sexually active in the past month, n (%)	39 (95.1)	41 (89.1)
Sexual behaviour, n (%)		
1 partner	33 (80.4)	37 (80.4)
>1 partners	8 (19.6)	9 (19.6)
Contraceptive use, n (%)		
Condom	29 (70.3)	34 (73.9)
Coitus interruptus	12 (29.7)	12 (26.1)
Start of CP history (months)^a^	18.7±4.28	19.1±3.99
Symptoms score at baseline^a^		
NIH-CPSI	24.9±2.1	25.5±3.0
IPSS	8.3±3.6	8.0±2.5
QoL	0.57±0.17	0.55±0.15
Clinical presentation, n (%)		
Dysuria	12 (29.2)	14 (30.4)
Urgency	4 (9.7)	5 (10.8)
Dysuria + frequency	6 (14.6)	5 (10.8)
Burning	7 (17.0)	9 (19.5)
Pain, n (%)		
Perineal	19 (46.4)	21 (45.6)
Scrotal	4 (9.7)	4 (8.7)
Suprapubic	8 (19.6)	9 (19.6)
Lower abdominal	10 (24.3)	12 (26.1)
Pain frequency, n (%)		
Daily	33 (80.4)	36 (78.2)
Weekly	8 (19.6)	10 (21.8)
Sexual Symptoms, n (%)		
ED	12 (29.2)	13 (28.2)
PE	7 (17.0)	6 (13.0)
ED + EP	4 (9.7)	4 (8.6)
CP/CPPS type, n (%)		
Type a	12 (29.2)	13 (28.2)
Type b	29 (70.8)	33 (71.8)

a Data are presented as the mean ± standard deviation.

CP, chronic prostatitis; NIH-CPSI, National Institutes of Health-Chronic Prostatitis Symptom Index; IPSS, International Prostate Symptom Score; QoL, Quality of Well-Being; CPPS, chronic pelvic pain syndrome; ED, erectile dysfunction; PE, premature ejaculation.

**Table II:** Questionnaire results at the follow-up visit.

Variable	DEPROX 500^®^ group	Ibuprofen group
NIH-CPSI (P<0.001)^a^	12.8±2.20	19.5±2.10
IPSS (P=0.87)^a^	7.6±1.58	8.00±2.81
QoL (P=0.002)^a^	0.69±0.10	0.59±0.18
Reduction in NIH-CPSI pain domain^b^, n (%)	31 (77.5)	19 (47.5)
Efficacy outcomes (NIH-CPSI pain domain)^a^,^c^	−4.36±0.51	−2.22±0.53
Efficacy outcomes (NIH-CPSI pain domain)^a^,^d^	−3.86±0.21	−2.10±0.20

a Data are presented as the mean ± standard deviation;

b pain, P<0.003;

c intention-to-treat analysis, treatment difference −2.41±0.51, P<0.001;

d per protocol analysis, treatment difference −1.76±0.22, P<0.001.

NIH-CPSI, National Institutes of Health-Chronic Prostatitis Symptom Index; IPSS, International Prostate Symptom Score; QoL, Quality of Well-Being.

**Table III:** Results of the sub-analysis on the basis of CP/CPPS type a or b.

Variable	DEPROX 500^®^ group	Ibuprofen group
Patients, n	40	40
Type a, n (%)	14 (35)	11 (27.5)
Type b, n (%)	29 (65)	32 (72.5)
NIH-CPSI^a^		
Type a	13.1±1.8	20.2±1.9
Type b	11.7±1.7	19.1±2.7
IPSS^a^		
Type a	7.9±0.9	7.9±3.1
Type b	7.4±1.5	8.0±2.2
QoL^a^		
Type a	0.61±0.3	0.57±0.2
Type b	0.70±0.1	0.60±0.1

a Data are presented as the mean ± standard deviation.

NIH-CPSI, National Institutes of Health-Chronic Prostatitis Symptom Index; IPSS, International Prostate Symptom Score; QoL, Quality of Well-Being; CP/CPPS, chronic prostatitis/chronic pelvic pain syndrome.
